# miR-491-3p is Downregulated in Retinoblastoma and Inhibit Tumor Cells Growth and Metastasis by Targeting SNN

**DOI:** 10.1007/s10528-020-10007-w

**Published:** 2020-10-23

**Authors:** Yang Hu, Ming Zhao, Li Li, Jie Ding, Yu-Min Gui, Tan-Wei Wei

**Affiliations:** Department of Ophthalmology, Puren Hospital of Wuhan University of Science and Technology, No.1 Benxi Road, Qingshan District, Wuhan, 430080 Hubei Province People’s Republic of China

**Keywords:** Retinoblastoma, miR-491-3p, Tumor growth, Metastasis, Epithelial–mesenchymal transition

## Abstract

Retinoblastoma (Rb) is the most common pediatric malignant tumor of the eyes. Previous studies demonstrated that miR-491-3p is downregulated in various cancers. However, its function in Rb remains unknown. A total of 15 pairs of primary Rb tissues and adjacent noncancerous tissues were collected. Quantitative real-time PCR (qRT-PCR) was used to investigate the expression profiles of miR-491-3p. qRT-PCR, western blotting and in situ immunocytochemistry were performed to investigate the expression profiles of epithelial–mesenchymal transition-related proteins (E-cadherin, Vimentin and N-cadherin) in Rb tissues and Rb cell lines as well as cell morphology. Cell proliferation was estimated by MTS and colony formation assays. Apoptosis was determined by FACS, cell migration and invasion were analyzed using transwell chambers. MiR-491-3p’s target genes were predicted using target gene prediction databases. The interplay between miR-491-3p and SNN was evaluated through dual luciferase reporter gene assay. MiR-491-3p was significantly downregulated in mixed collection of 15 pairs of Rb tissues and Rb cell lines. Overexpression of miR-491-3p enhanced apoptosis, and significantly suppressed proliferation, migration and invasion of Rb cells. In contrast, the present of miR-491-3p inhibitor showed reversed results which apoptosis decreased, while cell proliferation of ARPE-19 cells increased. In addition, miR-491-3p increased the expression of E-cadherin, and dramatically decreased the expression of Vimentin and N-cadherin in Rb tissues and Rb cell lines, noticeable changes in morphology, too, as cells became less cohesive and more adhering. We found out that SNN was the pairing target of miR-491-3p and result showed that miR-491-3p and SNN interacted with each other. We also found out that the effects of miR-491-3p were in Rb cells were almost entirely canceled out at the overexpression of SNN. Our findings collectively suggest that miR-491-3p is an important tumor suppressor in Rb, which inhibits tumor growth and metastasis in Rb. These implicate it may be explored as a new therapeutic target in Rb.

**Electronic supplementary material:**

The online version of this article (10.1007/s10528-020-10007-w) contains supplementary material, which is available to authorized users.

## Introduction

Retinoblastoma (Rb) is a form of cancer that rapidly develops from the immature cells of a retina, the light-detecting tissue of the eye. It is the most common primary malignant intraocular cancer in children, and it is almost exclusively found in young children (Neoplasms of the Eye 2003). The morbidity rate of Rb is about 1:15,000–1:20,000 in human infant (0–5 years). It is estimated that almost 9,000 new cases are annually reported worldwide (Ramirez-Ortiz et al. 2017). The Rb growth usually occurs under the retina and toward the vitreous cavity, result in the retinal detachment. In addition, it can spread to the central nervous system, and causes the death of patient (Fernandes et al. 2017). Hence, there is a dire need to explore the initiation and progression of Rb as well as its underlying mechanisms, which is helpful to develop more effective treatments.

MicroRNAs (miRNAs) are small noncoding single stranded RNAs and they regulate cellular and molecular processes, particularly gene expression by binding to complementary sequences in the 3′-untranslated region (3′-UTR) of target mRNAs. Through their transcriptional regulatory functions, miRNAs can control tumor proliferation, invasion and metastasis (Sethi et al. 2014a). Irregular expressions of miRNAs often contribute to the initiation and progression of cancers, for instance, Zhu et al. has demonstrated that miR-17-5p was able to enhance pancreatic cancer proliferation (Zhu et al. 2018), while Gao et al.’s study suggested that miRNA-223 may also play an essential part in both hematological malignancies and solid tumors (Gao et al. 2017). Extensive literatures also demonstrated that miRNAs play an important role in Rb. Researchers have found that miR-18a could inhibit tumorigenic potential of Rb while inhibition of miR-106b significantly increased the expression of the Rb genes (Busch et al. 2017; Samal et al. 2017). Additionally, miR-21 inhibitor suppressed the progression of Rb via the modulation of PTEN/PI3K/AKT pathway (Gui et al. 2016). In a previous literature, miR-491-3p was identified as a hypoxia-regulated miRNA (HRM) in Rb cells using microarray analysis (Xu et al. 2011). More importantly, miR-491-3p was already found playing a critical role in various cancers. In tongue cancer, mTORC2 downregulated miR-491-3p expression by inactivating FOXO1 (Zheng et al. 2015); while in osteosarcoma miR-491-3p functions as a tumor suppressor to attenuate the potential of growth and invasion by targeting TSPAN1 (Duan et al. 2017). Other researchers also found that miR-491-3p was able to attenuate multidrug resistance of hepatocellular carcinoma (Zhao et al. 2017). However, the biological role of miR-491-3p in Rb development remains elusive. EMT is a process by which epithelial cells lose their cell polarity and cell–cell adhesion and gain migratory and invasive properties to become mesenchymal stem cells; and it is a hallmark of initiation of metastasis in cancer progression. Using western blotting analysis and immunocytochemistry, we evaluated the expression levels of three EMT biomarkers: E-cadherin, Vimentin and N-cadherin, as well as morphology changes in Rb cells. Among them, E-cadherin is an epithelial cell marker, while Vimentin and N-cadherin are mesenchymal cell markers (Zeisberg and Neilson 2009).

Stannin (SNN) is a highly conserved vertebrate protein that consists of a single transmembrane helix, an unstructured linker domain, and a cytoplasmic domain. Previous study has revealed that it is closely linked to trimethyltin (TMT) toxicity and has played important role in cell apoptosis (Reese et al. 2005). Numerous studies have also indicated SNN plays the important role in tumor’s growth cycle (Reese et al. 2006). However, there is no study to examine the interaction between SNN and miR-491-3p and how this interaction can regulate the progression of tumor.

In this study, we first examined amount of miR-491-3p expression in Rb tissues and cells compared with normal, noncancerous tissues. Subsequently, we investigated the role of miR-491-3p in Rb cell proliferation, apoptosis, migration and invasion. Last, we used EMT (epithelial–mesenchymal transition) markers to test the role of miR-491-3p in Rb metastasis. Our results demonstrated the importance of miR-491-3p in regulating Rb development. This study was also the first to reveal miR-491-3p and SNN’s interaction to regulate proliferation, migration, invasion and apoptosis of Rb cell. This study provided novel insights into the biological function of miR-491-3p and showed that it may be a potential therapeutic target for Rb.

## Materials and Methods

### Tissue Samples

A total of 15 pairs of primary Rb tissues and adjacent noncancerous tissues were collected from the Wuhan University of Science and Technology Affiliated Hospital, Puren Hospital (Wuhan, China) between 2016 and 2017. The median age of patients was 2.5 years. Table 1 showed detailed information about the patients’ subphenotype, gender, age, tumor size, tumor stage and whether the patient has family history of retinoblastoma. The obtained tissues were immediately snap-frozen in liquid nitrogen and stored at − 80 °C. The diagnosis of Rb was confirmed by histopathological examination. All the cases were diagnosed and classified according to the International Classification of Retinoblastoma (ICRB). None of the subjects received any biotherapy, chemotherapy or radiotherapy treatment before recruitment to this study. All experiments were preformed according to the university’s human object use guidelines with approval from IRB (institution review board) and the hospital’s ethics committee. Signed informed consent from the participants or parent/legal guardian was obtained before the experiment.

**Table 1:** The count of clinical characteristics in retinoblastoma

Subphenotypes	Case (*N* = 15)
Gender	
Male	8
Female	7
Age (year)	
≤ 5	9
> 5	6
Tumor size (mm)	
≤ 15	7
> 15	8
Tumor stage	
I + II	4
III + IV	11
Family history	
Positive	10
Negative	5

### Cell Lines and Cell Culture

Human retinal pigment epithelium cell line ARPE-19 and Rb cell lines (Weri-Rb1 and Y79) were purchased from the American Type Culture Collection (Manassas, VA, USA). ARPE-19 cells were cultured in a 1:1 mixture of Dulbecco's modified Eagle's medium (DMEM) and Ham's F-12 medium (Invitrogen, Carlsbad, CA), supplemented with 10% fetal bovine serum (FBS, Omega Scientific, Tarzana, CA), 1% penicillin–streptomycin and 2 mM glutamine (Invitrogen, Carlsbad, CA), whereas Weri-Rb1 and Y79 cells were cultured in RPMI-1640 medium with 20% FBS, 1% penicillin–streptomycin and 2 mM glutamine (Invitrogen, Carlsbad, CA). All cell lines were cultured at 37 °C with 5% CO_2_.

### Cell Transfection

The miR-491-3p mimics (5′-AGTAGAAGGGAATCTTGCATAAG-3′) and its negative control (mimics NC) (5′-ACAGAGCTATAGATGTAAGTGAG-3′) were purchased from GenePharma Co., Ltd (Shanghai, China). miR-491-3p inhibitor (5′-CUUAUGCAAGAUUCCCUUCUAC-3′) and its negative control (inhibitor NC) ('5-GGUUCGUACGUACACUGUUCA-3′) were purchased from Sigma-Aldrich. Cells were transfected with miR-491-3p mimics, mimics NC, miR-491-3p inhibitor or inhibitor NC using Lipofectamine 2000 reagent (Invitrogen; Thermo Fisher Scientific, Inc.) according to the manufacturer instructions. Briefly, 5 μL Lipofectamine 2000 combined with miRNAs at a concentration of 30 nmol/L were incubated in Opti-MEM I reduced medium (Invitrogen) for 20 min prior to transfection. The mixture was then added into a 6-well plate with 5 × 10^4^ cells per well. Medium were changed to complete growth medium with 20% FBS after 6 h incubation at 37 °C. After transfection for 48 h, cells were harvested. Stannin (SNN) human tagged ORF clone lentiviral particle (oe-SNN), Lenti-ORF control particles (vector) and transfection reagent TurboFectin were obtained from OriGene and followed manufacturer’s preparation protocols.

### RNA Isolation and Quantitative Real-Time PCR (qRT-PCR)

Total RNAs was isolated by using a TRIzol Reagent (Invitrogen, Carlsbad, CA) according to the manufacturer’s instructions. The extracted RNA concentration and quantity were assayed using the NanoDrop ND-1000 Spectrophotometer (NanoDrop, Wilmington, DE). The RNA samples were reverse transcribed into complementary DNA (cDNA) using the TaqMan™ MicroRNA Reverse Transcription Kit (Applied Biosystems, Foster City, USA) following the manufacturer’s protocols. Quantitative real-time PCR (qRT-PCR) was executed by TaqMan Universal PCR Master Mix (Applied Biosystems, Foster City, USA). The amplification was monitored on the 7500 Fast Real-Time PCR System (Applied Biosystems, Foster City, USA), and the thermal cycling conditions were 95 °C for 5 min, followed by 40 cycles of 95 °C for 10 s and 60 °C for 45 s. U6 was used as an internal control. The following primers were used for the qRT-PCR: miR-491-3p forward primer, 5′-ATGCAAGATTCCCTTCTACAAA-3′ and reverse primer, 5′-CATGATCAGCTGGGCCAAGA-3′; U6 forward primer, 5′-CTCGCTTCGGCAGCACA-3′ and reverse primer, 5′-AACGCTTCACGAATTTGCGT-3′, SNN forward primer, 5′-AGTCAGAGGACGAGGAGAGCAT-3′ and reverse primer, 5′-ATCCTGGCTCAGCCGTGGACT-3′. Relative expression levels were evaluated using the $2^{{ - \Delta \Delta C_{{\text{t}}} }}$ method. All the reactions were performed in triplicate.

### MTS Assay

The proliferation of the transfected cells in each group was determined by using the MTS solution cell proliferation assay kits (Promega Corporation, WI, USA) according to the manufacturer’s instructions. The MTS assays were performed on the four consecutive time points (0 h, 24 h, 48 h and 72 h). The absorbance at 490 nm was read by SpectraMax M2 microplate reader (Molecular Devices, LLC, CA, USA), and then the cell proliferation curve was established. The experiment was independently executed three times.

### Soft Agar Colony Formation Assay

Transfected cells were prepared as a single cell suspension in complete DMEM media, and mixed with 0.6% (equal volume) low-melting point agarose (Sigma-Aldrich Co., St Louis, MO, USA). The mixture was laid on top of 0.6% solidified agarose in DMEM in 6-well plates (1 × 10^3^ cells/well). The growth medium was changed regularly every 3–4 days for 2 weeks. Cell colonies containing at least 50 cells were stained with crystal violet (Sigma-Aldrich Co., St Louis, MO, USA) and counted. The images were captured under a microscope (Leica Microsystems, Wetzlar, Germany) and analyzed. The experiment was performed in triplicate, and independently performed three times.

### Apoptosis Assay

Cell apoptosis was performed using the FITC-Annexin V Apoptosis Detection Kit I (BestBio, Shanghai, China) according to the manufacturer’s instructions. Briefly, the transfected Weri-Rb1 and Y79 Cells were resuspended at a density of 1 × 10^6^ cells/mL in PBS, then stained with FITC-Annexin V and propidium iodide (PI) for 15 min. The cells were analyzed using a cytoflex flow cytometer and Beckman CXP software (Beckman Coulter, Pasadena, CA, USA).

### Cell Migration and Invasion Assays

The cell migration and invasion assays were performed using the 8 μm pore size chamber inserts containing polyethylene terephthalate membranes (Corning Incorporated, Corning, NY, USA). For invasion assay, the transwell chambers were precoated with 30 μL 20% Matrigel (BD Biosciences, Franklin Lakes, NJ, USA) diluted in RPMI-1640 medium. A volume of 100 μL cells (0.5 × 10^6^ cells/mL) was resuspended in 200 μL RPMI-1640 medium without FBS, and seeded in the upper compartment of the chamber. In addition, 500 μL medium with 10% FBS was added into the lower compartment of the chamber. Following 24 h incubation, the cells attached on the upper surface were removed using cotton swabs. The migrated and invaded cells were counted and images were taken under an inverted microscope in five randomly selected visual fields (Leica Microsystems, Wetzlar, Germany). Both of the assays were repeated independently three times.

### Western Blotting

The protein was extracted from Rb tissues (from mixture of 15 Rb patients) and transfected Weri-Rb1 and Y79 cells using Radioimmunoprecipitation assay (RIPA) lysis buffer (Auragene Bioscience Co., Changsha, China) according to the manufacturer's protocols. The concentration of extracted proteins were determined by Bradford protein assay reagent (Beyotime Biotechnology, Suzhou, China).

The proteins were subjected to 12% SDS-PAGE (Bioworld Technology, Inc., St. Louis Park, MN, USA) and transferred to polyvinylidene difluoride membranes (PVDF) (Millipore, Bedford, MA, USA). The membranes were blocked with 5% nonfat milk in TBST buffer (Tris-buffered saline plus 0.05% Tween-20) for 2 h at room temperature, and then incubated with primary antibodies (E-cadherin (1:1500, ab15148, Abcam, China), Vimentin (1:1000, ab24525, Abcam, China), N-cadherin (1:2500, ab76057, Abcam, China), SNN (1:1000, ab121330, Abcam, China), GAPDH (1:5000, ab8245, Abcam, China)) overnight at 4 °C, washed three times with TBST and then incubated with a suitable secondary antibody [horseradish peroxidase-conjugated secondary antibody (ab7090, Abcam, China)] at room temperature. The membranes were visualized with enhanced chemiluminescence solution (Pierce, Thermo Fisher Scientific, Rockford, IL, USA) according to the manufacturer’s instructions. Images were captured using the FluorChem imaging system (Alpha Innotech, San Leandro, CA, USA), and densitometry analysis was performed using the Image J software. GAPDH was used as loading control.

### miRNA Target Prediction

Four publicly available databases were used for miRNA target prediction including Hoctar, miRDB, mirDIP and Targetscan. The prediction criteria were first 10th percentile of the ranked list for Hoctar (Gennarino et al. 2011), target score higher than 80 for miRDB (Wong and Wang 2015), “very high” and integrated score of higher than 0.5 for mirDIP (Tokar et al. 2018) and threshold was set to cumulative weighted context+ + score less than − 0.4 for Targetscan (Ma et al. 2018). The final prediction result was taken where these four prediction tools intersected. The result of the final prediction then be confirmed with luciferase reporter assay.

### Luciferase Reporter Assay

The full length 3′-UTR of SNN and the mutant derivative devoid of the miR-491-3p target site were amplified and cloned into psi-CHECK2 luciferase reporter vector to construct SNN 3′UTR luciferase reporter (SNN-WT) and target site mutation SNN luciferase reporter (SNN-MUT) respectively. The constructed nucleotide sequences of the plasmids were confirmed by DNA sequencing technique, and transfected with miR-493-3p mimics or mimics NC by Lipofectamine 2000 transfection reagent (Invitrogen) for 48 h. After that, luciferase reporter assays were carried out with Luciferase Reporter Assay kit (Promega) according to manufacturer protocol.

### In Situ Immunocytochemistry (ICC)

Cover slips was immersed in 2% H_2_O_2_ for 4 h and then washed 10 times with water and followed by ddH_2_O for 3 times. Cover slips was individually pull out and sterilized with flame, allowed to cool and placed in a container, then, coated with 100 μg/ml sterilized Poly-l-lysine solution for 5 min and rinsed with ddH_2_O for 3 times. Cells were seeded for overnight to the container. Media was removed and rinsed twice with 1XPBS at room temperature, and fixed in cool 4% paraformaldehyde in 1XPBS on ice for 15 min. After that, paraformaldehyde solution was removed and rinsed with 1XPBS 3 times at room temperature and the cells were permeabilized with 0.3% Triton X-100 in PBS for 15 min. 0.3% H_2_O_2_-methanol solution was added and incubated for 10 min after removing Triton X-100 solutions with PBS. After that, incubating solution was removed and incubated with 2% BSA in 1XPBS for 30 min at 37 °C to block nonspecific hybridization. Antibodies (anti-E-Cadherin; ab15148, anti-Vimentin; ab137321) were diluted in 1XPBS, 2% BSA according to manufacturer's instruction and applied to the cells on cover slips and incubated for 1 h at 37 °C. After that the incubating buffer was removed and rinsed 3 times with 1XPBS, then incubated with Alexa Fluor secondary antibodies (Abcam China) in 1XPBS, 2% BSA for 30 min at 37 °C. After the incubation, cover slips were rinsed with 1XPBS 4 times and immersed in DAB solution. Cover slips were closely monitor and rinsed with 1XPBS as soon as the cells turned brown. Cells then were counterstained in hematoxylin for 10 min, washed in 1XPBS, immersed in 1% acid-alcohol and rinsed with tab water. The coverslips were then washed with PBS and were mounted onto glass slides for imaging.

### Statistical Analyses

All statistical analyses were performed using SPSS 18.0 software (SPSS Inc., Chicago, IL, USA). Data are represented as mean ± standard deviation (SD), and statistical significance was set at *p* < 0.05. Differences between two or multiple groups were analyzed by Student's t-tests or one-way analysis of variance (ANOVA) followed with Tukey’s post-hoc analysis, respectively.

## Results

### miR-491-3p is Downregulated and Epithelial–Mesenchymal Transition is Activated in Rb Tissues and Rb Cell Lines

**Fig. 1:**
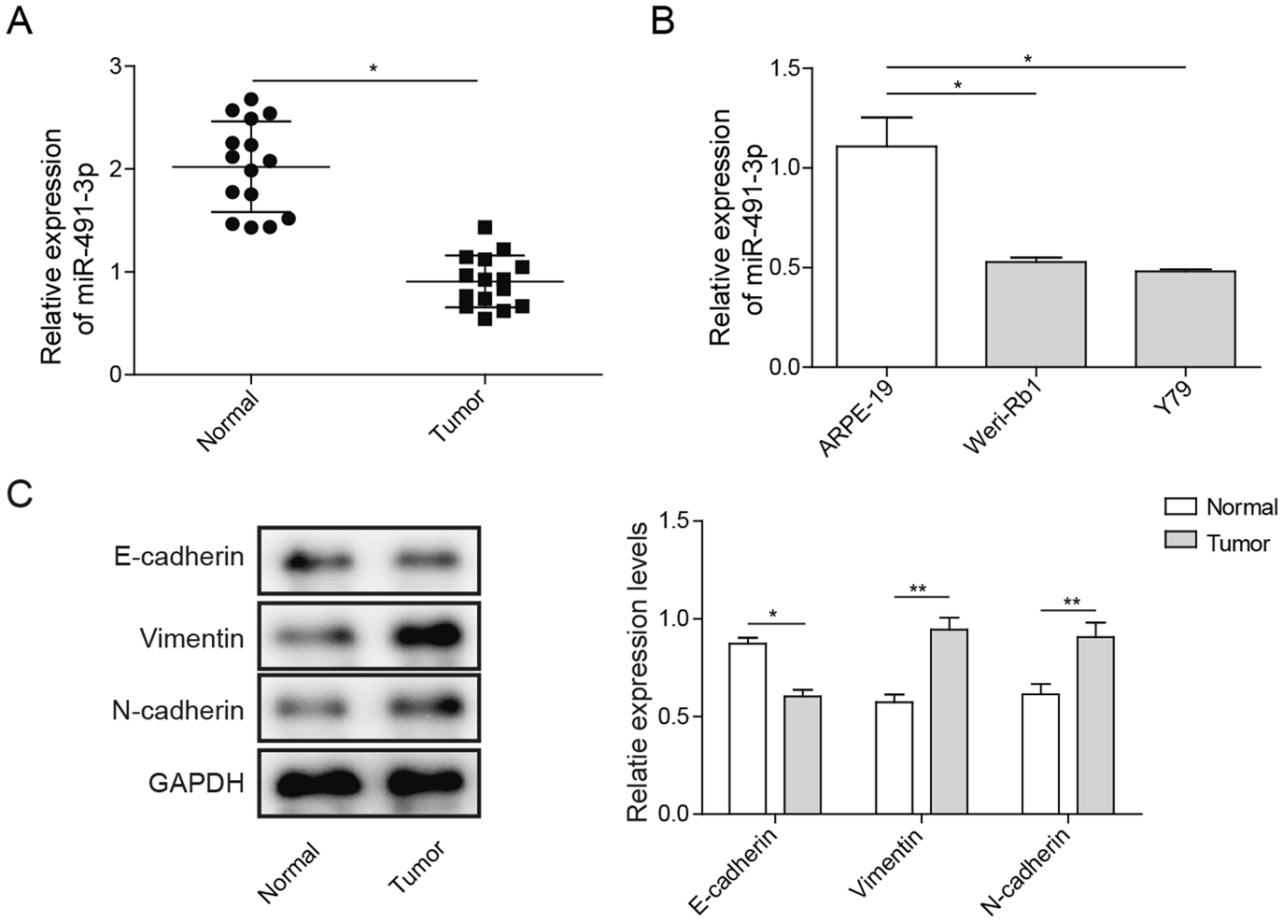
Expression profiles of miR-491-3p in Rb tissues and Rb cell lines and epithelial–mesenchymal transition occurs. **a** qRT-PCR analysis of miR-491-3p expression in Rb tissues and adjacent noncancerous tissues. **b** qRT-PCR analysis of miR-491-3p expression in Rb cell lines (Weri-Rb1 and Y79) compared to normal human retinal pigment epithelial cell line ARPE-19. **c** Western blot of the expression of EMT-related proteins—E-cadherin, vimentin and N-cadherin in normal ARPE-19 or Rb tissue. All results are from three independent experiments. Bars represent mean ± SD (*n* = 3, *p < 0.05)

First, qRT-PCR was performed to evaluate the expression of miR-491-3p in Rb and adjacent noncancerous tissues. As shown in Fig. 1a, the expressions of miR-491-3p were significantly downregulated (almost 50%) in Rb tissues compared to noncancerous tissues. Second, we also examined miR-491-3p expression in Rb cell lines (Weri-Rb1 and Y79), and found that miR-491-3p was significantly inhibited nearly 50% in Weri-Rb1 and Y79 cell lines compared to ARPE-19 cell line (Fig. 1b). Our study results demonstrated that miR-491-3p was downregulated in Rb tissues and Rb cell lines***, ***which may suggest miR-491-3p may play an important role Rb development. As shown in Fig. 1c, protein expression of the E-cadherin from the 15 Rb patients was significantly decreased in tumors, whereas N-cadherin and Vimentin expressions were significantly increased as compared to normal ARPE-19 cell. The results indicated that miR-491-3p was downregulated and EMT was activated in Rb tissues and Rb cell lines.

### Overexpression of miR-491-3p Suppresses Cell Proliferation of Rb Cells In Vitro

**Fig. 2:**
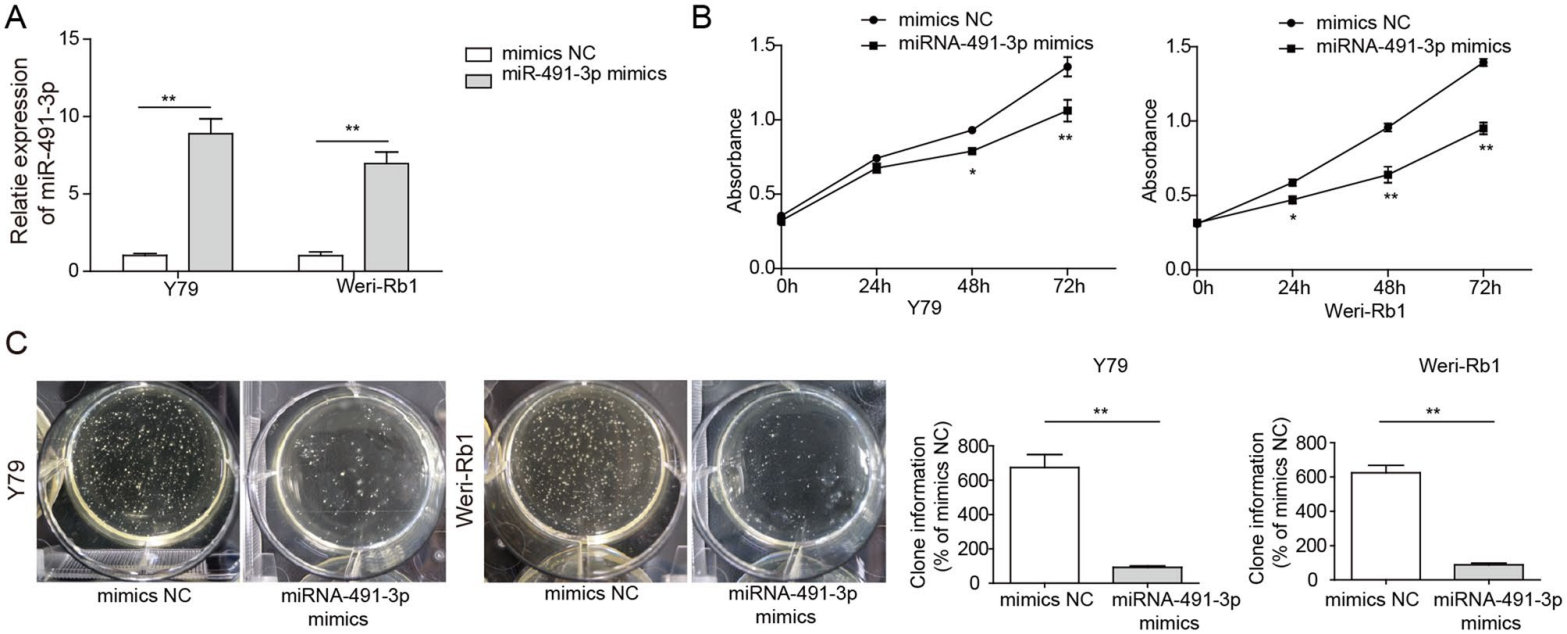
Overexpression of miR-491-3p suppressed cell proliferation in Rb cell lines. **a** qRT-PCR analysis of miR-491-3p expression in Weri-Rb1 and Y79 cells transfected with miR-491-3p mimics, mimics NC. **b** MTS assay of cell proliferation of Y79 and Weri-Rb1 cells transfected with miR-491-3p mimics, mimics NC. **c** Colony formation assay of cell proliferation in Y79 and Weri-Rb1 cells transfected with miR-491-3p mimics, mimics NC. Left image is clone formation on the culture dish; right is the number of clones quantitatively analyzed. All results are from three independent experiments. Bars represent mean ± SD (*n* = 3, *p < 0.05)

To understand the function of miR-491-3p in cell proliferation of Rb, Y79 and Weri-Rb1 cells were transfected with either miR-491-3p mimics, mimics NC, subsequently, the proliferation activities were measured using MTS and colony formation assays. We first confirmed the effectiveness of miR-491-3p overexpression by miR-491-3p mimics as shown in Fig. 2a. The miR-491-3p levels of cells transfected with miR-491-3p mimics were about 12 times more than cells transfected with mimics NC (Fig. 2a). Further, MTS assay results demonstrated that miR-491-3p overexpression significantly suppressed cell proliferation in Y79 cells and in Weri-Rb1 cells from 24 to 72 h (Fig. 2b). The soft agar colony formation assay showed that colonies of both Rb cell lines were significantly inhibited after miR-491-3p mimics transfection compared to the colony formation of both cell lines transfected with mimics NC (Fig. 2c). The results suggested that miR-491-3p was critical for Rb cell proliferation.

### Overexpression of miR-491-3p Suppresses Migration and Invasion of Rb Cells In Vitro

**Fig. 3:**
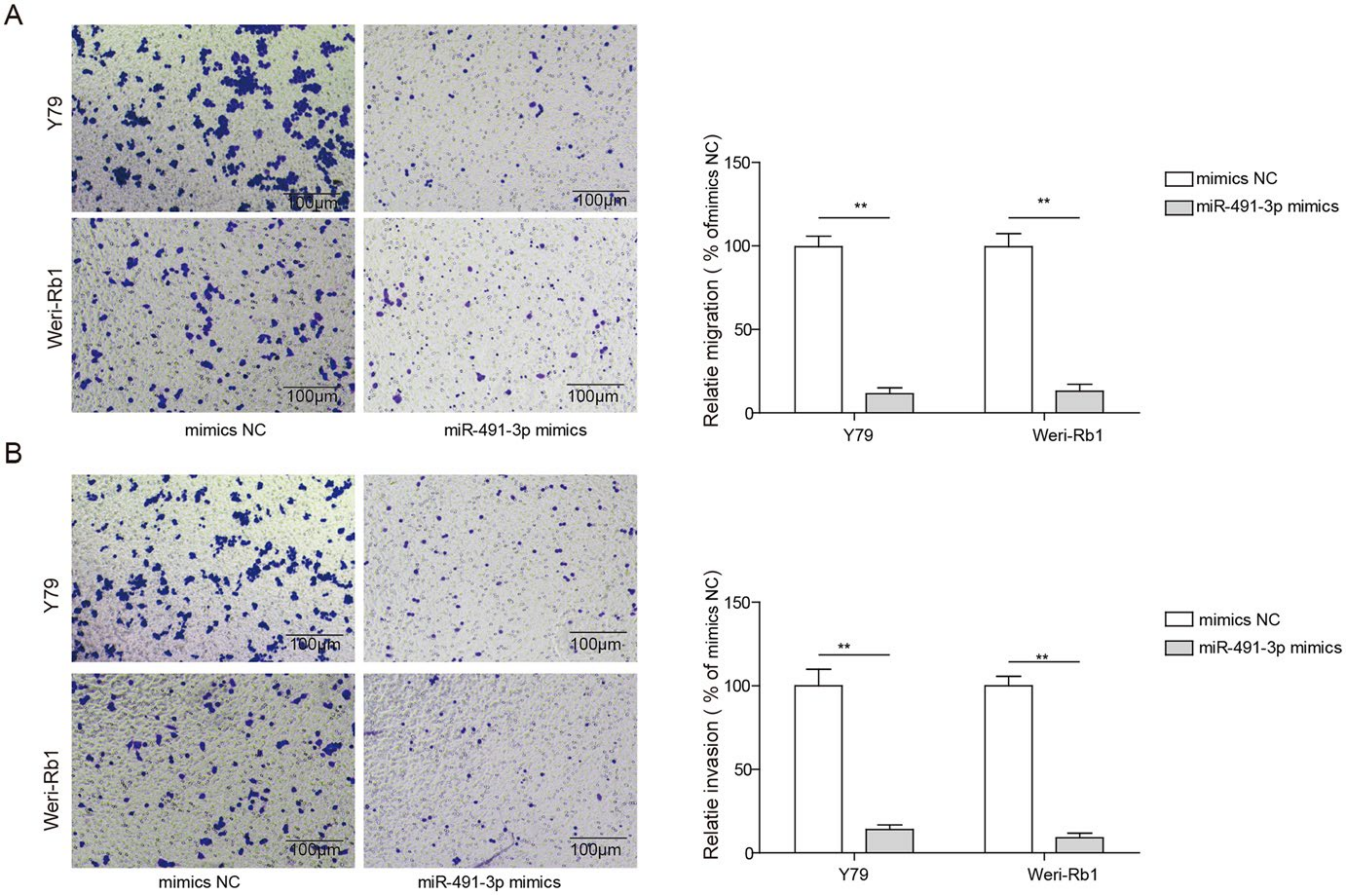
Overexpression of miR-491-3p decreases cell migration and invasion in Rb cell lines. **a** Left image is staining of cell migration for cell transfected with miR-491-3p mimics, mimics NC under the inverted microscope (magnification, × 100); right image is statistical analysis of left image. **b** Left image is staining of cell invasion for cell transfected with miR-491-3p mimics, mimics NC under the inverted microscope (magnification, × 100); right image is statistical analysis of left image. All results are from three independent experiments. Bars represent mean ± SD (*n* = 3, *p < 0.05)

We also elucidated the role of miR-491-3p in cell migration and invasion using transwell assays. After transfected with either miR-491-3p mimics or mimics NC, the migrated cells were decreased nearly 75% for the Y79 cell line and 65% for Weri-Rb1 cell line transfected with miR-491-3p mimics as compared to cells transfected with mimics NC (Fig. 3a). Furthermore, we also found that the percentages of invaded cells were markedly decreased more than 50% in both Rb cell lines after transfected with miR-491-3p mimics as compared to the cells transfected miR-491-3p NC (Fig. 3b). The results suggested that overexpression of miR-491-3p reduced the migration and invasion capacity of Rb cells.

### Overexpression of miR-491-3p Enhances Cell Apoptosis and Affects EMT Process of Rb Cells In Vitro

**Fig. 4:**
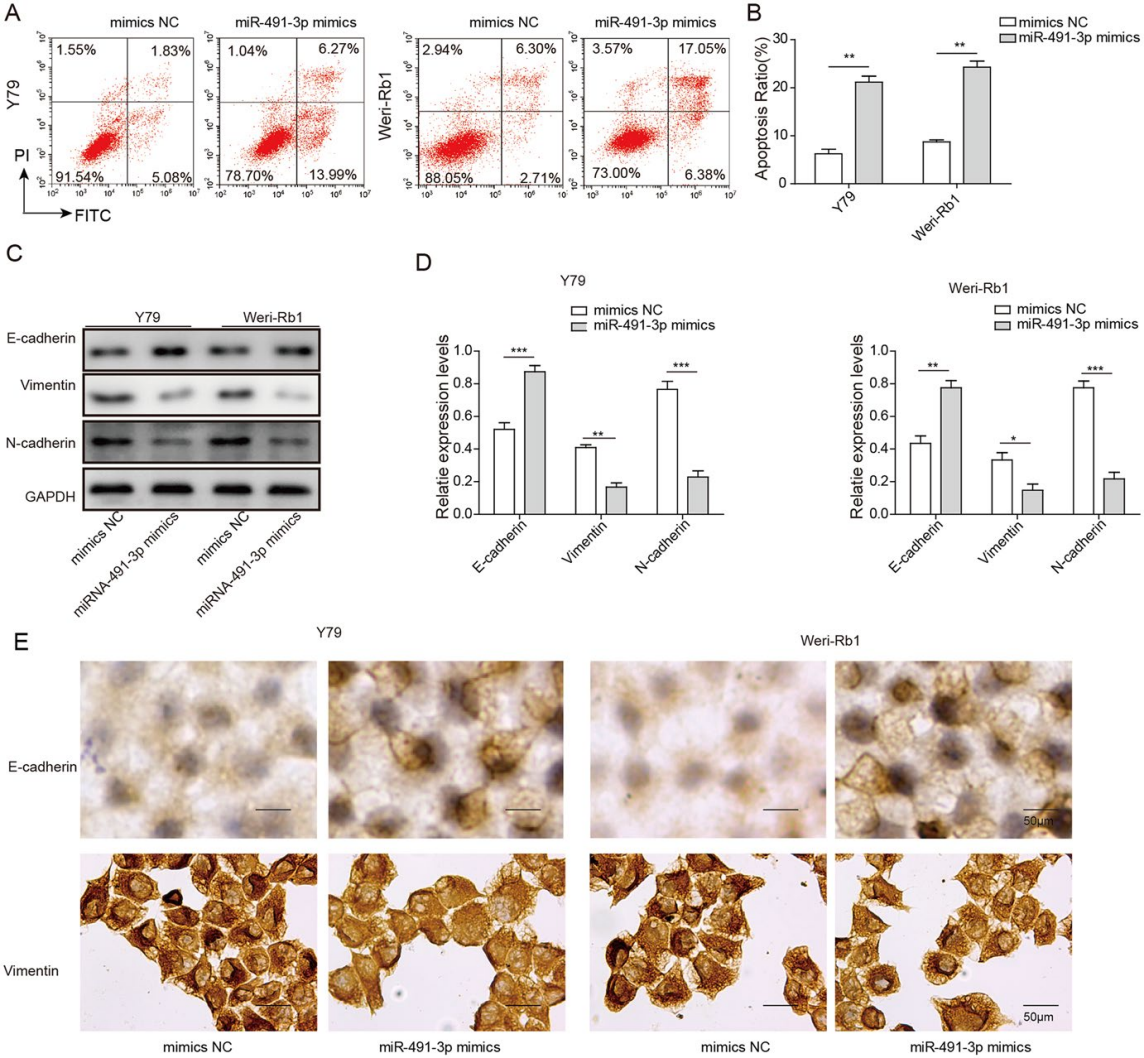
Overexpression of miR-491-3p induced apoptosis and inhibited EMT in Rb cell lines. **a** FACS assay of apoptosis in Y79 and Weri-Rb1 cells transfected with miR-491-3p mimics, mimics NC, NC. **b** Apoptosis ratio was quantitatively analyzed. All results are from three independent experiments. **c**, **d** After Rb cell line transfected with miR-491-3p mimics, mimics NC, E-cadherin protein level was increased, while vimentin and N-cadherin level were decreased compared to cells transfected with mimics NC. **e** In situ immunocytochemistry of Rb cell line transfected with miR-491-3p mimics, mimics NC (magnification, × 400). Left image showed the morphology changes of cells, right image is statistical analysis of left image. All results are from three independent experiments. Bars represent mean ± SD (*n* = 3, *p < 0.05)

To further examine the role of miR-491-3p in Rb, we performed FACS assay to explore mechanism of miR-491-3p regulation of Rb cell growth. We found that overexpression of miR-491-3p promoted cell apoptosis in both Y79 and Weri-Rb1 cells (Fig. 4a). With miR-491-3p mimics transfection, cell apoptosis increased approximately two-fold in both Y79 and Weri-Rb1 cells compared with cells transfected with mimics NC (Fig. 4a, b). In combination with previous results, we found that miR-491-3p inhibits Rb cell proliferation, colony formation and enhances cell apoptosis. Our study also indicated that EMT process was reversed when miR-491-3p is overexpressed. As shown in Fig. 4c, d, E-cadherin was significantly increased, while N-cadherin and Vimentin were significantly decreased after Rb cells was transfected with miR-491-3p mimics. Our ICC results were also aligned with the result from western blotting, which E-cadherin was seen increased but both N-cadherin and Vimentin were significantly decreased in miR-491-3p mimics group as compared to miR-491-3p NC group. Morphology changes were noticeable in Y79 and Weri-Rb1 cells, too, both cell cultures cohesion had decreased and became more adhering in miR-491-3p mimics group as compared to miR-491-3p NC group which was more cohesive and spheroidal (Fig. 4e). The results suggested that overexpression of miR-491-3p inhibited migration and invasion of Rb cells through regulating EMT.

### Low Expression of miR-491-3p Inhibits Apoptosis and Enhances the EMT Process of ARPE-19 Cells

To further verify the effect of miR-491-3p on retinoblastoma. Another piece of data to include miR-491-3p inhibitor and inhibitor NC in the normal cells were conducted. ARPE-19 cells transfected with miR-491-3p inhibitor had lower level of miR-491-3p expression than inhibitor NC (Fig. S1A). Further, MTS assay results demonstrated that proliferation in ARPE-19 cells from 24 to 72 h had increased when ARPE-19 cells were transfected with miR-491-3p inhibitor as compared to cells transfected with inhibitor NC (Fig. S1B). We found that cells transfected with miR-491-3p inhibitors showed further decreased in cell apoptosis as compared to inhibitor NC (Fig. S1C). As shown in Fig. S1D, E-cadherin was significantly decreased, while N-cadherin and Vimentin were significantly increased after Rb cells was transfected with miR-491-3p inhibitor as compared to the cells transfected with inhibitor NC. In combination with previous results, we found that low expression of miR-491-3p inhibits apoptosis and enhances the EMT process of ARPE-19 cells.

### Stannin is Targeted by miR-491-3p and Upregulated In Vivo and In Vitro

**Fig. 5:**
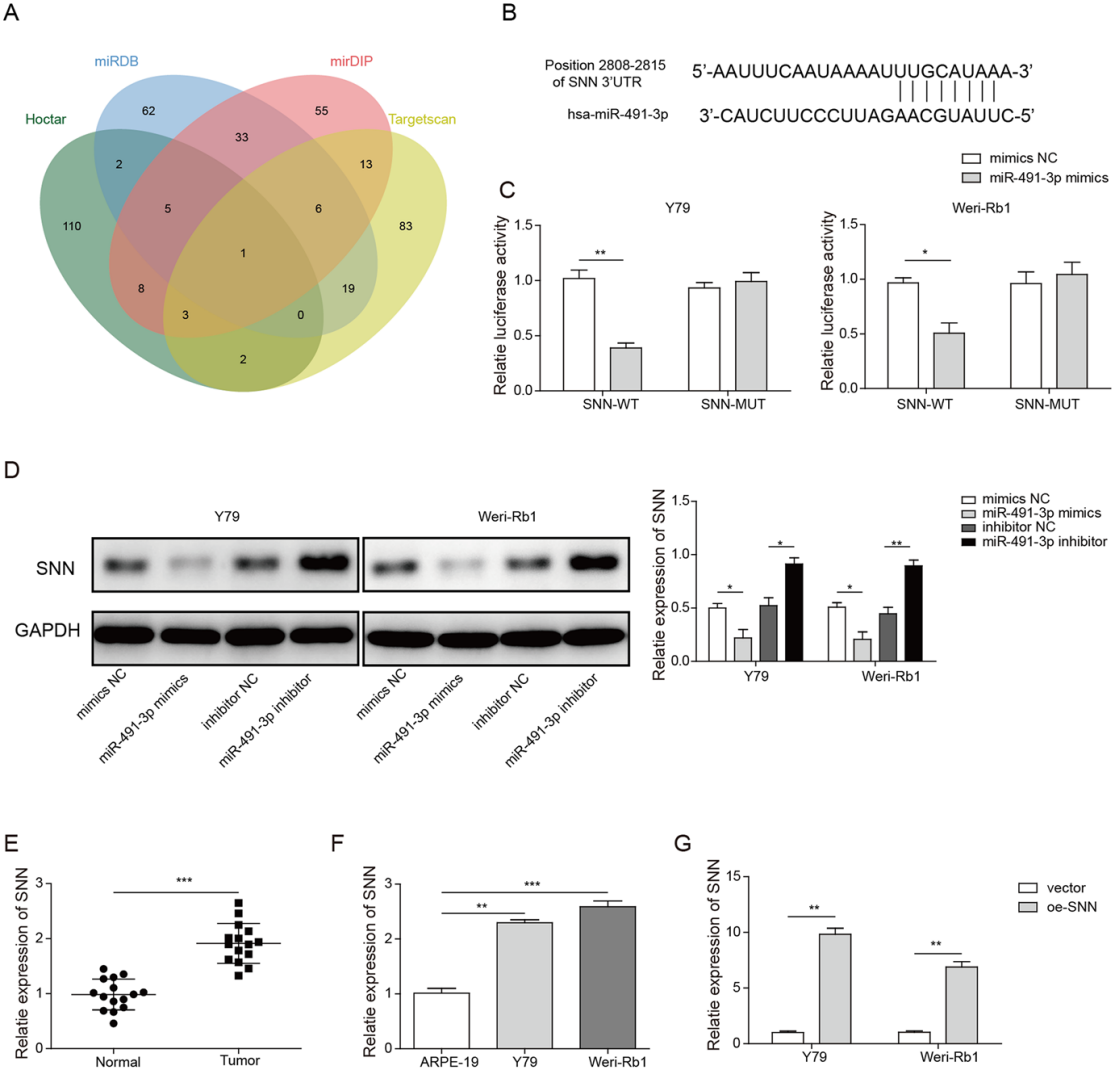
Stannin is targeted by miR-491-3p and upregulated in vivo and in vitro. **a** The prediction result was taken from the intersection of the 4 miRNA target prediction databases. **b** Prediction of pairing location of miRNA miR-491-3p to mRNA SNN. **c** Luciferase reporter assay of Y79 or Weri-Rb1 cells with SNN-WT or SNN-MUT transfected with mimic NC or miR-491-3p mimics. All results are from three independent experiments. **d** Western blotting of the expression of SNN in Y79 and Weri-Rb1 cells transfected with mimics NC, miR-491-3p mimics. **e** RT-qPCR analysis of expression of SNN in Rb tissues and adjacent noncancerous tissues. All results are from three independent experiments. **f** RT-qPCR analysis of expression of SNN in ARPE-19, Weri-Rb1 or Y79 cells. All results are from three independent experiments. **g** RT-qPCR analysis of transfection rate of oe-SNN in the Y79 or Weri-Rb1 cells. All results are from three independent experiments. Bars represent mean ± SD (*n* = 3, *p < 0.05)

Using four publicly available miRNA databases Hoctar, miRDB, mirDIP and Targetscan, we predicted that miR-491-3p pairs with mRNA Stannin (SNN) at position 2808–2815. Between 127 and 131 genes came out as results after we applied our prediction criteria to each of these prediction tool, but only 1 gene, SNN was shared by all the results of these tools (Fig. 5a, b). The results from our luciferase reporter assay have confirmed that SNN-WT interacted with miR-491-3p mimics as the activity of the SNN-WT in both Y79 and Weri-Rb1 cells were significantly reduced in the present of miR-491-3p mimics as compared to mimics NC. The result also showed that miR-491-3p did not affect SNN-MUT. This result indicated that miR-491-3p only interacted with wild-type SNN by binding to the specific site of the gene, and miR-491-3p has no effect on SNN-MUT as the binding site was mutated (Fig. 5c). Our western blot results also demonstrated that SNN was downregulated with the overexpression of miR-493-3p, but was upregulated when miR-493-3p was suppressed (Fig. 5d). RT-qPCR, our study further revealed that SNN was overexpressed in the both Rb cells Weri-Rb1 and Y79 as compared to normal and ARPE-19 cells (Fig. 5e, f). We were also able to confirm that transfection rate of SNN to the Weri-Rb1 and Y79 was very high as the SNN expression was more than ten-fold higher as compare to the vector group (Fig. 5g). The results indicated that miR-491-3p targeted and regulated SNN in both Weri-Rb1 and Y79 cells.

### SNN Eliminates Promotion of miR-491-3p on Rb Cells Proliferation, Migration and Invasion

**Fig. 6:**
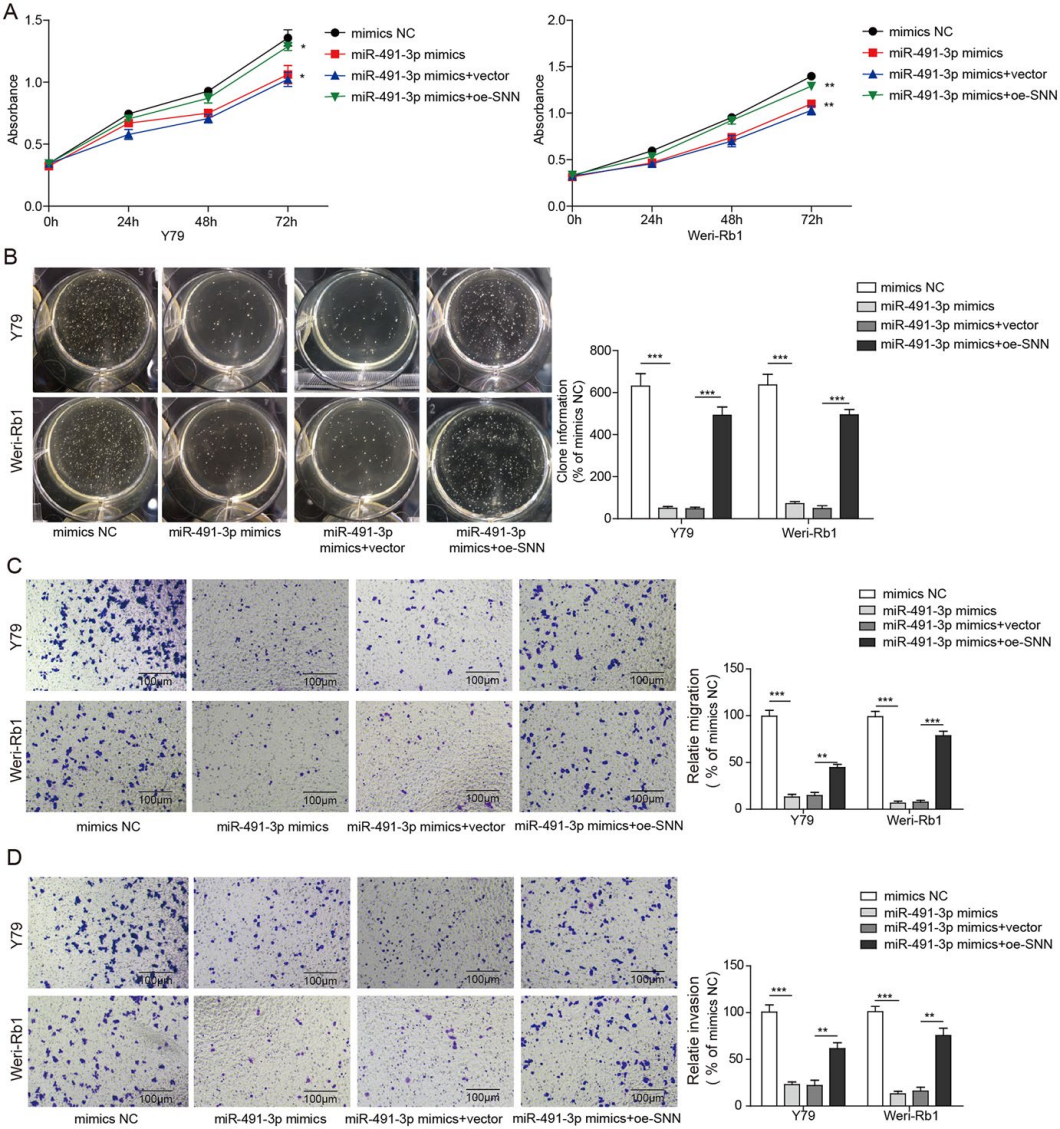
SNN eliminates promotion of miR-491-3p on Rb cells proliferation, migration and invasion. **a** MTS assay of cell proliferation of Y79 and Weri-Rb1 cells, or transfected with miR-491-3p mimics, miR-491-3p + vector or miR-491-3p + oe-SNN. **b** Colony formation assay of cell proliferation in Y79 and Weri-Rb1 cells, or transfected with miR-491-3p mimics, miR-491-3p + vector or miR-491-3p + oe-SNN. Left images are clone formation on the culture dish of Y79 and Weri-Rb1 cells, or transfected with miR-491-3p mimics, miR-491-3p + vector or miR-491-3p + oe-SNN; right chart is the number of clones quantitatively analyzed. All results are from three independent experiments. **c** Left images are staining of cell migration of Y79 and Weri-Rb1 cells, or transfected with miR-491-3p mimics, miR-491-3p + vector or miR-491-3p + oe-SNN under inverted microscope (magnification, × 100); right chart is statistical analysis of these clone cultures. **d** Left images are staining of cell invasion of Y79 and Weri-Rb1 cells, or transfected with miR-491-3p mimics, miR-491-3p + vector or miR-491-3p + oe-SNN under inverted microscope (magnification, × 100); right chart is statistical analysis of these clone cultures. All results are from three independent experiments. Bars represent mean ± SD (*n* = 3, *p < 0.05)

To understand the relationship of SNN to miR-491-3p in Rb, Y79 and Weri-Rb1 cells were transfected with either mimics NC, miR-491-3p mimics, miR-491-3p mimics + vector or miR-491-3p mimics + oe-SNN; subsequently, the proliferation activities were measured using MTS and colony formation assays. The results demonstrated that overexpression of the SNN cancel out the cell proliferation suppression effects of the miR-491-3p mimics in Weri-Rb1 and Y79 cells from 24 to 72 h (Fig. 6a). The soft agar colony formation assay showed that colonies of both Rb cell lines were significantly inhibited after miR-491-3p mimics transfection, but then elevated by the overexpression of SNN (Fig. 6b). The role of SNN in cell migration and invasion were also examined with transwell assays. The migrated Rb cells were decrease significantly after transfected with miR-491-3p mimics were subsequently promoted by the overexpression of SNN. The effect of miR-491-3p mimics was almost canceled out entirely by oe-SNN (Fig. 6c, d). Our result showed that SNN eliminated the effect of proliferation, migration and invasion those promoted by miR-491-3p in Rb cells.

### SNN Eliminates Influence of miR-491-3p on Apoptosis and EMT of Rb Cells

**Fig. 7:**
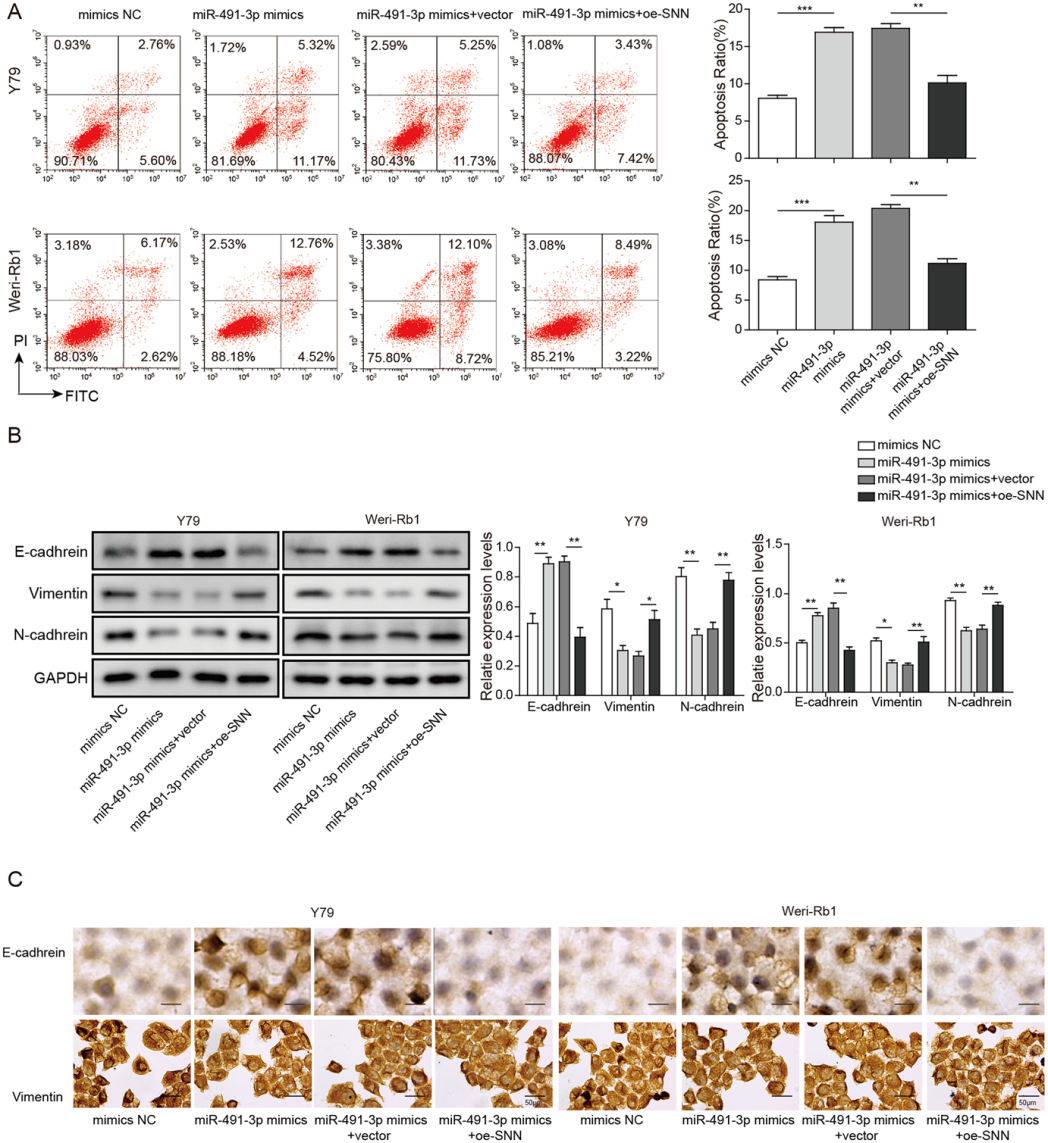
SNN eliminates influence of miR-491-3p on apoptosis and EMT of Rb cells. **a** Left images are the FACS assay of apoptosis in Y79 and Weri-Rb1 cells, or transfected with miR-491-3p mimics, miR-491-3p + vector or miR-491-3p + oe-SNN. Right chart is the quantitative analysis of the apoptosis rate of these images. **b** E-cadherin, vimentin and N-cadherin protein levels in Y79 and Weri-Rb1 cells, or transfected with miR-491-3p mimics, miR-491-3p + vector or miR-491-3p + oe-SNN. **c** In situ immunocytochemistry of Rb cell line transfected with miR-491-3p mimics, miR-491-3p + vector or miR-491-3p + oe-SNN (magnification, × 400). Left image showed the morphology changes of cells, right image is statistical analysis of left image. All results are from three independent experiments. Bars represent mean ± SD (*n* = 3, *p < 0.05)

To examine effect of SNN in Rb cell apoptosis and its involvement in the EMT process, we performed FACS assay and western blotting in present study. The results showed that overexpression of the SNN inhibited the enhancement of the miR-491-3p mimics made to the Rb cells apoptosis. The apoptosis rates of both Weri-Rb1 and Y79 cells were similar to mimics NC groups when miR-491-3p mimics were co-transfected with oe-SNN (Fig. 7a). Western blotting’s results showed similar trend, E-cadherin protein expression was significantly increased with the present of miR-491-3p mimics, but subsequently decreased after oe-SNN was transfected to the Rb cells. Vice versa, N-cadherin and Vimentin expressions were significantly decreased by miR-491-3p mimics, and subsequently increased with the transfection of oe-SNN (Fig. 7b). Our ICC result also confirmed similar trends, where protein expression of E-cadherin was increased, while N-cadherin and Vimentin’s expression were decreased with the present of miR-491-3p mimics, and oe-SNN reversed the effects. Cells cultures morphology changes were noticeable, too, cell cohesion decreased and cell became adhering in miR-491-3p mimics group, while cells were more cohesive and spheroidal in oe-SNN group (Fig. 7c). The results implied that the EMT process was inhibited by miR-491-3p but restored by the overexpression of SNN in the Rb development.

## Discussion

Rb is the deadliest intraocular malignant tumor in the retina of children and its prevalence has increased worldwide. Numerous studies showed that miRNAs play critical roles in tumor pathogenesis (Gao et al. 2017; Sethi et al. 2014b). Therefore, miRNAs are considered to be effective diagnostic and prognostic biomarkers or therapeutic target. Many chemical drugs exert their therapeutic affects through regulating and targeting various miRNAs in tumor (Salarinia et al. 2016). Additionally, downregulation of miRNAs are often associated with progression of tumor cells (Busch et al. 2017; Samal et al. 2017). Recently, many researchers demonstrated that miR-491-3p is downregulated in different tumors, and functions as a tumor inhibitor in the tumorigenesis of several malignancies by affecting multiple genes (Duan et al. 2017; Zhao et al. 2017; Zheng et al. 2015). However, prior to the present study, the role of miR-491-3p in Rb remains unclear. In the present study, we showed that overexpression miR-491-3p in Rb cells can suppress proliferation and induce apoptosis as well as promote cell migration and invasion. In additional, we also found that epithelial marker E-cadherin expression was increased, while mesenchymal markers N-cadherin and Vimentin expressions were decreased, cells became less cohesive and more adhering after miR-491-3p overexpression, suggesting EMT in Rb cells was regulated by miR-491-3p.

Many researchers have reported that the expression of miRNAs were downregulated in various tumor tissues (Drusco and Croce 2017; Han Li and Chen 2015). While in Rb, expressions of a number of miRNAs such as miRs-382, -486-3p, -129-3p, and -129-5p were suppressed in primary human Rb tissues and cell lines (Golabchi et al. 2018; Singh et al. 2016). It was identified that miR-491-3p expression was frequently decreased in osteosarcoma tissues and osteosarcoma cell lines, which functions as a tumor suppressor to attenuate the potential of growth and invasion by targeting TSPAN1 (Duan et al. 2017). The present study consistently demonstrates miR-491-3p expression is significantly downregulated in Rb tissues and Rb cell lines when compared with adjacent noncancerous tissues and ARPE-19 cells.

Extensive evidence has shown that overexpression of certain miRNAs can suppress tumor cell proliferation and enhances cell apoptosis in nearly all cancer types, such as skin cancer, lung cancer and cervical cancer (Li et al. 2018a; Ross et al. 2017; Vannini et al. 2018). Similarly, miRNAs also play important role in Rb. For example, miR-21 inhibited the cell proliferation via the modulation the cell apoptotic in Rb (Gui et al. 2016; Song et al. 2017). More importantly, miR-491-3p was also demonstrated to modulate tumor cell apoptosis and proliferation. Duan et al. found that as a tumor suppressor, restored miR-491-3p expression suppressed the growth of osteosarcoma cells (Duan et al. 2017). miR-491-3p also involved in tongue cancer and hepatocellular carcinoma (Zhao et al. 2017; Zheng et al. 2015). In this study, our findings showed that overexpression of miR-491-3p induces Rb cell apoptosis, and subsequently inhibits cell proliferation are consistent previous reports listed above. The results suggest that miR-491-3p is a tumor suppressor in Rb.

Additionally, we also showed that miR-491-3p can inhibit cell migration, invasion and EMT in Rb cells and tissues. EMT is a crucial step in cancer invasion and metastasis, in which cells lose their epithelial characteristics, and are replaced by a mesenchymal phenotype such as reduced cell–cell adhesion as well as increased migratory and invasive properties (Shao et al. 2017; Thiery 2002). In details, we found that E-cadherin expression was increased, while vimentin and N-cadherin expressions were decreased after overexpressing miR-491-3p in Rb cells. Epithelial marker E-cadherin is a type of Ca^2+^-dependent transmembrane glycoproteins regulates cell–cell adhesion. A deficiency of E-cadherin reduces the cell adhesion and promotes the cell migration and invasion in tumors. E-cadherin is also correlated with the prognosis of Rb, lower E-cadherin generally indicates bad prognosis (Shao et al. 2017). Vimentin and N-cadherin are mesenchymal markers indicating the initiation of cancer metastasis. Consistence to our results, a number of studies showed that miRNAs may regulate EMT in a variety of cancers. A group of researchers reported that miR-302b inhibits tumorigenesis by targeting EphA2 via EMT signaling cascade in gastric cancer (Huang et al. 2017), and others found that miR-125b-5p can function as a tumor suppressor in esophageal squamous cell carcinoma (Mei et al. 2017). In the present study, our data confirmed that miR-491-3p could inhibit migration and invasion in Rb cells through regulating EMT, and it may be a novel targeted therapy for Rb. According to published research, miR-491-3p directly targeted insulin-like growth factor 1 receptor (IGF1R) and MKK7, a mitogen-activated protein kinase, to suppress tumor metastasis (Kumar et al. 2017; Sakai et al. 2014). MiR-491-3p was also reported to directly target RhoC and FZD4 to suppress bladder tumor cell mobility (Ueno et al. 2012). Finally miR-491-3p was reported to target YTHDF2, a methylation of the N6 position of adenosine (m6A) reader protein, and mitotic arrest deficient-2 (Mad2) to affect tumor cell proliferation (Li et al. 2018b; Tambe et al. 2016). We believe that they work similarly in Rb tumor genesis and progression. In future study, more experiments will be conducted in order to explore the interaction among miR-491-3p, YTHDF2 and Mad2 in tumor suppression in Rb.

The present study found out the miR-491-3p targeted mRNA SNN. Target site-mutated SNN did not interact with miR-491-3p in our study has confirmed our prediction, which was consistence with similar approach in other studies (Cheng et al. 2019; Dong et al. 2020). But there was a pity that we were unable to include SSN-MUT in the rest of our experiments certainly was an unfortunate limitation to our study that was due to time and cost constraint, but we will certainly revisit this limitation in our future study. SNN has been shown to play an important role in the toxic effect of organotin (Davidson et al. 2004) and endosomal maturation (Pueyo et al. 2016). Study of SNN in oncology, however, is scarce, but Reese et al. demonstrated that protein kinase C regulates Tumor Necrosis Factor-Alpha (TNF-α) induced SNN expression (Reese et al. 2005), and our study is the first to reveal miR-491-3p and SNN’s interaction to regulate proliferation, migration, invasion and apoptosis of Rb cell. We also know that SNN is highly involved in MAPK signaling pathways (Davidson et al. 2005) and MAPK signaling pathway has shown to be important in some cancers (Dhillon et al. 2007). This is consistence to our study which showed the proliferation, migration and invasion of the Rb cells correspond to the over expression of SNN, and it silences the effects of miR-491-3p. Our study has showed that overexpression of SNN promotes EMT process, too. The results suggested that SNN may be linked to cell growth and proliferation by regulating EMT process via MAPK signaling pathway. Summarizing all the above studies, we speculate miR-491-3p—SNN link may have effect in other tumors, but need further investigation. However, the mechanism of the mRNA SNN’s involvement in the promotion of the Rb cells was not included in the present study. We do hope the present study may provide a renew interest for the future research.

In conclusion, miR-491-3p was markedly downregulated in Rb tissues and Rb cells. It inhibited tumor growth by inducing cell apoptosis, and suppressed the cell proliferation, cell migration and invasion of Rb cells. In additional, miR-491-3p dramatically increased the expression of E-cadherin and decreased the expression of Vimentin and N-cadherin in Rb cell lines, suggesting that it was involved in EMT of Rbs. Our study will provide novel insights into miR-491-3p as a tumor suppressor in Rb, and it could be a therapeutic target for Rb. However, the underlying mechanism of how miR-491-3p regulates Rb cell EMT markers and other bioactivities is still dimness. Thus, further study is needed to elucidate the entire cellular and molecular pathway of miR-491-3p in Rb. Additionally, we also planned in vivo animal experiments to confirm our in vitro results in future.

## Electronic supplementary material

Below is the link to the electronic supplementary material.

Supplementary file1 (TIF 744 kb)
Figure S1. Low expression of miR-491-3p inhibits apoptosis and enhances the EMT process of ARPE-19 cells. A: qRT-PCR analysis of miR-491-3p expression in ARPE-19 cells transfected with miR-491-3p inhibitor or inhibitor NC; B: MTS assay of cell proliferation of ARPE-19 cells transfected with miR-491-3p inhibitor or inhibitor NC; C: FACS assay of apoptosis in ARPE-19 cells transfected with miR-491-3p inhibitor or inhibitor NC. D: Western blot of the expression of EMT related proteins in ARPE-19 cells. All results are from three independent experiments. Bars represent mean ± SD (n = 3, * p < 0.05).

## Data Availability

All statistical analyses were performed using SPSS 18.0 software (SPSS Inc., Chicago, IL, USA).

## Abbreviations

Rb: Retinoblastoma
miRNAs: MicroRNAs
HRM: Hypoxia-regulated miRNA
EMT: Epithelial–mesenchymal transition
ICRB: International Classification of Retinoblastoma
NC: Negative control
SNN: Stannin
cDNA: Complementary DNA
RIPA: Radioimmunoprecipitation assay
PVDF: Polyvinylidene difluoride membranes
ANOVA: Analysis of variance
