# Supplementary figures and images for: miR-491-3p is Downregulated in Retinoblastoma and Inhibit Tumor Cells Growth and Metastasis by Targeting SNN
Source: Biochem Genet. 2020 Oct 23;59(2):453–74. doi: 10.1007/s10528-020-10007-w (PMC7946698; doi:10.1007/s10528-020-10007-w)

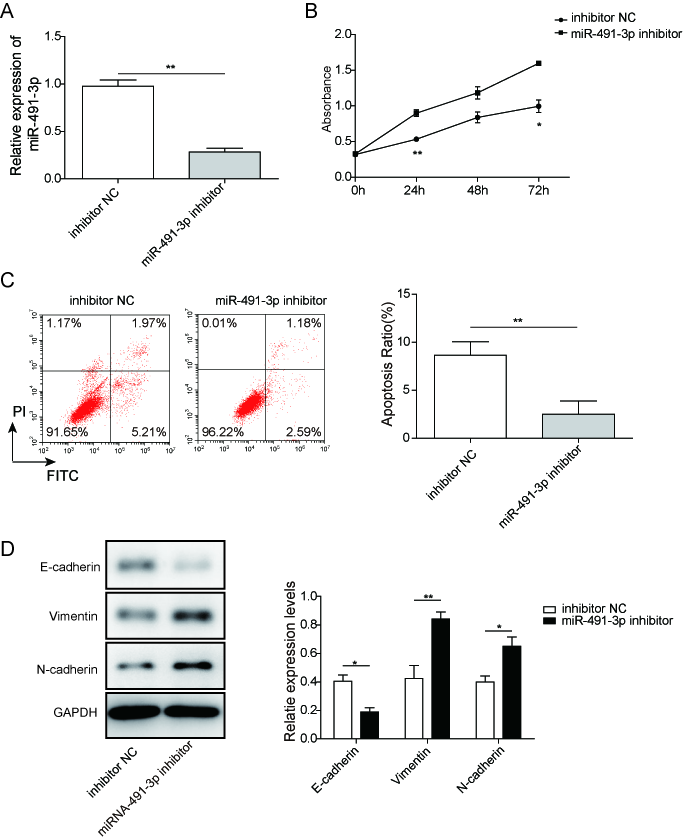

Supplement: Supplementary file 1 — Supplementary file1 (TIF 744 kb) Figure S1. Low expression of miR-491-3p inhibits apoptosis and enhances the EMT process of ARPE-19 cells. A: qRT-PCR analysis of miR-491-3p expression in ARPE-19 cells transfected with miR-491-3p inhibitor or inhibitor NC; B: MTS assay of cell proliferation of ARPE-19 cells transfected with miR-491-3p inhibitor or inhibitor NC; C: FACS assay of apoptosis in ARPE-19 cells transfected with miR-491-3p inhibitor or inhibitor NC. D: Western blot of the expression of EMT related proteins in ARPE-19 cells. All results are from three independent experiments. Bars represent mean ± SD (n = 3, * p < 0.05). [file 10528_2020_10007_MOESM1_ESM.tif]
